# Lecture Introductory to the Seventh Annual Course of Lectures, in Atlanta Medical College

**Published:** 1861-06

**Authors:** H. W. Brown

**Affiliations:** Prof. of Anatomy


ATLANTA

Medical and Surgical Journal.

Vol. VI.] JUNE, 1861. [No. 10

### ORIGINAL COMMUNICATIONS.

### ARTICLE I.

*Lecture introductory to the Seventh Course of Lectures in Atlanta Medical College.* By H. W. Brown, M. D., Prof. of Anatomy.

Ladies and Gentlemen: Custom has made it necessary on occasions such as the present, when, in any of our Institutions of learning, the regular didactic course is beginning, to introduce the exercises by some general prefatory remarks, bearing more or less relation to, and explanatory of the future course; such an Inaugural is peculiarly appropriate to a course of Medical instruction. The young mind is just entering upon the exploration of vast and illimitable fields, to tread the labyrinthian paths of the most comprehensive Science known to human investigation; to soar in those regions of mental conflict, which have tried the swift wing of genius to its utmost: have occupied the mightiest intellects till the sands of life were spent, and commands, now, the loftiest and most sublime operations of the human understanding. Here, too, youth from various sections of our country, are, without previous acquaintance with teacher or subject taught, suddenly convoked to enter upon an arduous, and, to most, untried course of study—a course not only intended to impart to them the principles of future action, but to furnish the whole armour of future conflicts; to place in their hands those weapons which, to wield aright, is to bless mankind—unskillfully, to leave wreck and ruin, death and devastation in their wake. Again, too, instead of the younger pilgrims in the paths of Science, whose defective judgments require control, often without appeal, here we propose to direct mind already well developed; judgment for the most part reliable; and understanding rapidly approaching maturity; youth giving freshness and vigor only to the aspirations of early manhood.

The Temple of Science, inviting as she may be to the hopeful observer, massive and eternal in her proportions, and the steadfastness of her butments and arches; wealth, fame, honor, immortality, beckoning from her wide extended portals; the accumulated light of ages playing o'er her towering dome, and culminating in brilliance around her Heaven-directed spire, is yet rugged in approach, and inaccessible to any save the laborious and patient devotee at her shrine. A life-time service of unremitting toil, fair Science claims of her followers; of those who would win her laurels—while untold pleasures thicken around the pathway, in increasing proportions, as the distance vanishes, and we near her fane.

This day a decision must be made or confirmed, for a life-time, by many, if not all who gather around our teaching; a decision pregnant with all the evil consequences of misdirected effort, or replete with joys to lighten the burden of time, and light our pathway to eternal rest. Will not all join me, then, in pronouncing this an interesting occasion? The pleasures and interest of which are not a little enhanced by this respectable concourse of our worthy citizens. Though various the motives which may prompt them to join us, we cannot fail to express our grateful appreciation of their manifest interest in all that pertains to our young and prospering Institution; nor especially to thank the fairer and more attractive portion of our audience for their encouraging smiles, and that tacit welcome to the stranger, read in many a sparkling eye. Indeed, gentlemen, the discharge of one pleasant duty this morning has been rendered almost unnecessary, by the flattering presence of so many bright faces here to greet you.

It is mine, Gentlemen of the Class, to express to each and all of you the pleasure experienced by the Faculty of this Institution, in thus meeting you. We have said to you, come; from every section of our much-loved South, come, where Southern hands are outstretched to greet you; where Southern hearts, beating in unison with your own, guarantee that genial hospitality we know well how to appreciate.

You are here, and this community, I feel free to say, extend to the worthy, the civilities and hospitalities of their homes. The Trustees of this Institution, ever ready and willing to serve our great cause, hail your coming with joy and gladness, and for each one of these, (the Faculty,) I promise without hesitation, all in their power, that warm hearts can inspire, a community of interests secure, or a profound sense of weighty responsibility may incite to make your stay with us profitable and pleasant—your future efforts honorable and useful—your eternal future blissful. With this high commission our Faculty have honored me to-day, and while I proceed with heart-felt anxiety to whisper words of counsel and encouragement, such as the occasion may suggest, I cannot forbear expressing to them my deep sense of the honor and confidence implied, only equaled by the consciousness of inability on my part to do them justice.

We can certainly do much, young gentlemen—you much more—towards the accomplishment of those great aims, which we hope have influenced you in coming to this temple of Medical Science. The great work which many of you now begin, demands at your hands all your energies, all of patient toil, animated by devoted love, and strengthened by determined will, that you can bring to bear. You are engaging to serve and defend a cause coextensive with, and involving, more or less, the good or ill, happiness or wo, of human life—its duration only ceasing in the last great catastrophe which shall call forth the sleeping dust of ages, animate the dry bones in the valley of death, and wrap our earth in her shroud of consuming flame. You are entering upon the study of Man—man as an animal, as a vegetable even; as a most complex and beautiful machine; as the living, breathing, moving, self-sustaining and controling wonder and climax of the created universe; Man physically, mentally, morally—the epitomé of Nature—the microcosm in whose embrace the treasures of creative power, wisdom and benificence reveal themselves to the faithful inquirer after Nature's truth—always the counterpart and supporter of God's revealed truth, when rightly understood.

The work of a life-time is before you; ay! even from an elevated stand-point of scientific attainment, we hear the sad confession—

"We look back on our life and find it near gone,
Unfinished e'en the task of a day;
Shrink from the thought of how little is done,
Shake hands with old Time and away."

Of what consequence, then, that we begin aright; that we properly understand and appreciate the duties of to-day!

—the only time we can claim as our own. To-morrow may never come—that day of promise, "in which fools reform, and the lazy do all their work." The truth loses nothing by repetition, that "time lost can never be reclaimed;" and I would impress indelibly on the minds of all, that duty neglected to-day can only be discharged in the future by sacrificing some other obligation, perhaps equally urgent.

Rather than make you an elaborate speech or essay, I would foreshadow, therefore, the duties immediately at hand, and offer a few suggestions by the way-side, which I trust may not be entirely useless.

Those departments of Medical Study upon which you now enter, you will surely learn to class under two heads, viz: those which are fundamental and demonstrative; next, those secondary and inferential. The first will require your earliest attention, for upon your understanding of these rests your entire future as Medical men. If you would be established, as upon a rock; if you would make every step in your course of study pleasant and satisfactory; if you would grow in thirst, and relish for knowledge, as you gain capacity to attain; if you desire that reputation which will be lasting, reflecting credit upon your instructors, and securing to your own brow unfading laurels; indeed, if you would be Men, display that determined courage which knows no fail, in meeting and mastering those trying studies, which lie at the very threshold of Medical inquiry.

The more difficult the task, the more value and glory in the attainment. Anatomy greets your first approach with clear and unmistakable revelations of our material organization. The assemblage of organs, active and passive, presents their various but established relations; the different *tissues* or textures composing them, (their very mention, a tribute of praise to the imperishable name of Bichat!) claim your careful scrutiny; at once the key and elucidation of the (at one time) confusing subject, Inflammation—explaining to the eye of the Anatomist, the various results of the one operating cause. Here, calling to our aid the microscope, we challenge competion in variety and beauty of revealed truth. Now, in the reticular structure of more permanent tissue, she displays, in mathematical relations, the delicate column, and the sustaining arch where levity and strength are requisite. Again, that wonderful system of vessels! miraculously accurate in capacity, and relation to the forces, bearing forward the crimson tide of life with its myriads of ambassadors, and to the delicate structures, which, in their turn, receive and impart, in accordance with mutual demand, vieing with the rainbow in soft impressive tints.

Indeed might we protract these exercises without limits in the bare mention of mysterious truth and beauty, opening up, as from an inexhaustible mine, to the increasing delight of the devoted student. Yes, gentlemen, dry and uninteresting as often appears to the tryo in Medicine, the minutiæ of Anatomical detail, yet I promise you, studied in the light of its important relations, each fact brought out, properly interrogated and classified—each part carefully consulted as to its vital connections and uses, with the earnestness of true philosophical inquiry, the dullest mind will be aroused to lively interest; pleasure will be added to interest in due time, that even the phlegmatic, prosy, musty, moth-eaten pedant may be inspired with the ecstasy of delight.

Just here, let me enter a protest against an opinion too often expressed by those, for whose sentiments (in general,) we entertain most profound respect. 'Tis often said by those occupying high places in the World of Letters—too often, as

I have heard, caught up and repeated by Ministers of the Gospel of Christ, expounders of the Apostolic word—that there is a tendency in the study of Anatomy to lead the heart from God and the established truths of His Bible, to supplant pure, spiritual religion, and set up in lieu thereof a base and degrading materialism. Now, I deny the proposition, and protest against it as a gross injustice to this department of Science, the result of imperfect investigation, and more faulty reasoning, as diametrically opposite to the teachings of the great inspired Apostle Paul. I believe it easy to establish that in those great minds, ornaments to this department of Science, while they darkened the moral atmosphere in which they moved, we have coincidence merely; moral sentiments perverted by the circumstances, and teaching in which they took root and developed, seeking that support in Nature too often predetermined; and establishing no more against the study of Anatomy, than the erring lights of the pulpit to condemn Bible investigation.

Is it not evident, from the most casual investigation of animal forms, that purpose, adaptation and design are there clearly expressed with the existence of laws as clearly influencing their action, announcing a designer and law-giver, in every manifestation, *infinitely* superior to human conception?

But what need we more than the langauge of St. Paul in refutation of this unjust charge, who tells us, "the invisible things of God are clearly seen, *being understood by the things that are made?*" In so many words, stating the fact that the existence and character of God may be established by His works.

We are brought next, by pleasing and insensible gradations, to consider the vital and healthy action of the various parts—their functions as they are called, and their multiplying relations. Yes, Physiology, almost inseperable from Anatomy, demands an early consideration, exhibiting the forces, principles and laws concerned in the phenomena of life —at once the agents and results of this vital machinery. Here is required careful scrutiny, patient, logical, inductive reasoning; while, to the speculative, is opened wide the door for the wildest flights of fancy.

The Materia Medica, among the fundamental branches, should be met and mastered early; Nature and Art here exposing the rich treasures of that great Arsenal, (the collective work of centuries,) from which you select the armour of intelligent Medical service.

Chemistry, generally too much neglected, demands of the Medical man in this day of improvement, his most flattering attentions. She teaches him the laws governing matter in all its unions and reunions, to constitute the forms he has examined; prepares his remedial agents; and elucidates most, if not all, the complex phenomena which characterize the vital organism. These, with the study of Pathological Anatomy, or those changes which disease effects in the various organic structures, whether by slow, steady and insidious approach, or as an overwhelming, devouring, devastating enemy of life—mantling bright hope with the vestures of dark and crushing despair, should, ay! must be first understood before the inferential or practical can be profitably engaged in. Yet, how often do we see the young men of our ranks hurrying over these with the reckless haste of a Jehu, to reach and grapple with the practical branches, Surgery, Practice, &c.? It is a stigma and reproach on the intelligence and understanding of our young men, a burning shame upon our profession, to witness the deep concern manifest in gathering scraps of practical medicine with the degrading, futile purpose to convert a rational reasoning brain or mind, God-like in its legitimate and enobling action, into a mere receptacle of heterogenous, irrelevant facts; deposing reason from her throne for the usurping genius of a gazetteer or farmer's almanac, without attempting a basis upon which may safely rest the explanation of the simplest Medicinal or Physiological action. How often heard the bitter tirade against Quacks, and Quackery, while Ignorance, culpable ignorance, in our own ranks, which should mantle our cheek with shame, furnishing plausible arguments to the mouth of Empericism, defacing the lines of destinction between us, meets little rebuke, no condemnation!

I regard with feelings more nearly akin to respect the veritable Quack, whose defective education, mental and moral, has made ignorance his shield and fortification, than the unreflecting routinist, pandering only to the whims and caprices of his patrons for their filthy lucre.

I trust you will not misunderstand me. You cannot believe your humble speaker, however much he may deplore our own defects, would defend, or be for one moment the apologist of the bold, unscrupulous, unfeeling monster of Ignorance and Vice, whose whole armour is impudence, whose entire course of blighting deception is marked by the seed of physical suffering; moral deformity and untimely decay springing thick in his track. No, no. But let us rather, regarding quacks as a necessary evil in consequence of defective knowledge of ourselves, springing as noxious weeds from the slough and scum of general ignorance on these subjects, lay broad and deep our own foundations, make fast the expanding roots of our noble tree of Medical Knowledge, that its sturdy, time-honored trunk, and majestic boughs, already claiming universal respect, as they stretch out to protect and bless mankind, may cast off the blighting parasites which cumber and annoy its weaker branches, by the overshadowing verdure of its own healthy foliage.

Now that you have such an acquaintance with these branches which must constitute the basis of all successful medical study; now that you have proper insight into the wonderful mechanism, laws and actions of the animal, more restricted still the human economy, you have done much in the right direction, in compliance with the precept of a Pagan Philosophy, to "know thyself." You are only then prepared to recognise correct data, logical induction and the reliable influence of the higher, more practical branches; only then prepared to consider symptomatology, or indications of present disease; the magic sympathy which morbid action establishes between remote parts; the demands which tortured nature makes for skillful aid to check the ravages of disease, or modest request to be let alone in performance of her easy and appropriate work.

We rejoice to see a constantly increasing desire on the part of our medical men for proper self-cultivation. We are proud to see the increasing facilities offered; schools and colleges dotting the whole surface of our fertile, happy, Heaven-blessed South, introducing such text-books, often, to male and female, as will prepare the rising generations for proper understanding upon these subjects; thereby moving the Medical student to more enlarged and thorough work of preparation, and making broad and distinct to every eye the lines of demarkation between the ignorant trifler with life's best interests, whether claiming the M. D. or not, and the man of true scientific attainment, whose profound sense of weighty responsibility clothes every profession with modesty.

Again we congratulate you, gentlemen, on entering a field of inquiry already surveyed, opened and producing well, but still prepared and promising the most bountiful yield to your honest toil.

The tangle and brushwood; the gloomy forest surrounding and overshadowing Medical Science for many centuries, admitting only here and there a ray of light, as it yielded to the sturdy, yet often rude, stroke of a Galen, Hippocrates, Paracelsus, Sydenham, Harvey, Hunter, Brussais and others, by the light of a Baconian Philosophy, opened up, cleared and converted to an artistically arranged garden, only awaiting the systematic labor of the husbandman to blossom as the rose. Much has been done already; much remains to cheer your future efforts, of bright promise.

Pharmacy presents you with the beautiful yet potent alkaloid, in lieu of the bulky and nauseous draught. The protracted illness of weeks is checked and dissipated in so many days. The heated chamber, foul air and steaming drinks give place to ventilation, pure air and refreshing draughts. The parching tongue is moistened; the famishing thirst quenched; the demands of Nature, as common sense satisfied by the purest, best and most acceptable of all drinks—cold water. The once tortured victim, by recent surgical improvements, is made to leap for joy, as the cripple of old under the Divine touch of the Great Physician, and the distorted countenance, reflecting the anguish of unspeakable pangs, by a simple apparatus, radiant with kindling hope and suffering relieved. But many improvements are yet necessary to make our armories complete and satisfactory; many are the questions of propriety in the use of agents now generally employed. Disease still baffles too often the most assiduous and skillful efforts of the physician; and while 'tis not likely we shall ever attain that perfection which the eye of the philanthropist and father of American Medicine saw in the dim future of hope; yet, I feel, we all know of improvements constantly made; that we are daily becoming more familiar with Disease, and better acquainted with the means of cure. The race with the fell monster is still going on; but we have gained much in distance and confidence; and now to you, gentlemen, beginning with all the strength and vigor of young hope, with additional means and appliances furnished by the accumulated experience of the profession, we look with cheering confidence to push onward—gain the victory—wear the crown.

But, that your efforts may be successful, see to it that proper habits of thought and action are early established, while your minds yet retain plasticity, and your physical man is yet responsive to the high behests of the inward monitor. Habit, remember, is second nature. But while all readily assent to the proposition, how few, judging from the tenor and results of life, properly appreciate its vital truth! Did time allow, I fancy it would not be unprofitable to discuss at some length the claims of habit to our attention. But that we may incite to investigation and reflection, rather than fatigue you with any elaboration, I determine to present but a few thoughts, which, as seed scattered in good soil, may spring up in future to the production of a bounteous and profitable harvest.

Man—in his triple nature, as physical, intellectual and moral agent, the union and relations of these has been the subject of profound thought since the world began—yet remains the puzzling enigmatical source of speculative investigation, in many respects, as when the human mind first awaked to consciousness of self-existence. But the study of human life, in the light of his phenomena, individually and collectively, has given us much clearer insight to his nature, relations, duties and destinies. We have learned, by tracing effects to their proper causes, to perceive far beyond the apparent, the real.

So we find Custom, dependent on accident or circumstance providentially surrounding, begets habit, inclination or disposition, often counter to natural instinct as reason, fettering body and mind, unconsciously, it may be, but with the same destructive certainty as the tightening coils of the reptilian monster betoken death to his victim, though his fears are quieted by delusive promises, and the warm, hissing breath lulls with the lie of Eden—"Thou shalt not surely die." Why is it so? How is it the nauseous drug becomes a source of pleasure, often a necessity? Why is the offensive draught sought after, relished, demanded, in exchange for health, comfort, even life? The wine-cup pressed eagerly to cravings lips, by fingers already trembling at the approach of speedy dissolution? Why the repulsive scene now pleasant, aye, bewitching? Why the panting haste of the frenzied mob to glut its fiendish cravings at the rack and guillotine, and lap with brutal delight the blood of beauty and innocence? The force of habit—the result of long-continued thought and action favoring these unnatural developments. But how; and why such antecedents produce such consequences? brings us to recognise the important truth which underlies and explains all, viz: That every action is attended with organic, structural, molecular change; that life itself is but the action of this vital machinery, responsive to the inspiriting touch of that mysterious, etherial, eternal, indestructible essence, the Soul; that every normal action, so resulting, is followed by waste and elimination of particles forming the structure, and the acquisition of new atoms to repair and make anew the tissue. Again: That within certain limits, exercise or action of parts increases their nutrition, growth and capacity for action over and beyond the attendant loss. These are physiological truths, recognised, too, as manifested in the limb of the racer, the arm of the blacksmith, and the easy, elastic step of the danseuse. The opposite is seen, when inactivity induces waste without corresponding nutrition, in the flabby, attenuated limb, the pale cheek, whose capillaries, from long disuse, refuse a just response to cheering breath of morn, choke back the crimson tide of life and beauty, and drape the hallowed precincts of youth and loveliness with the sad insignia of premature decay. Oh! how long shall wasting indolence and blighting inactivity curse our race, squander our fair heritage and desolate the very temples of purity and love?

Nor is habit less worthy our consideration in its controlling power over the operations of the mental or intellectual man. Mind being but the manifest action of material brain, excited by impressions without and controlled by immortal essence within; the intellectual powers, therefore, are clearly dependent upon the brain as the instrument or medium upon which, and through which, God-like spirit shall act in communication with matter. The resultant phenomena we recognise as mind, or else we can form no conception of it. The same laws controlling physical development elsewhere, are here alike manifest, nourishing, strengthening and expanding (within certain limits) in just accordance with exercise of the several parts of acting brain.

Now, therefore, if you would have Mind ready, active, powerful, tune the instrument; bring out the several keys to their full tune and strength by invigorating use, that, under the magic touch of the willing essence, its power, compass and harmony may captivate while it controls. It is by proper habits of thinking we have ready power to think. Do you suppose, the active, thriving merchant became such by birth or accident? Oh, no. Continued effort, in obedience to sovereign will, has established habit—made it easy and natural that he should perceive quickly, judge aright and act promptly. Nor is it chance that clothes the attenuated form with dignity—secures respect—gives persuasive power and eloquence to the passionless lip; virtuous emotion to the heart, and patriotic fire to the peerless eye of Georgia's honored statesman—piercing while it illuminates! No; but the early formation of such habits as evolve such character. What has converted the heads of the canting, pharisaical, sensation preachers of New England into fit tenements for all the damning heresies and isms that curse our land?—prepared their hearts to abandon God's holy truth for usurping lies and abominable abstractions, whose source is Hell—whose end is death? What, but long-continued habits of thought, established by Cromwell, the bigot, tyrant, fiend—perpetuated through a long line of Puritan fathers, and now developing brains whose unnatural, unholy emanations obscure the Sun of Righteousness, mutilate the Word, now destroy the fairest and best of Earth's Governments?

In a word, gentlemen, if you would have your future lives successful and happy; if you would be loved, revered and respected by all; if you would be truly great physically, intellectually and morally, now, while the pliant fibres of the twig yield readily to the moulding will, determine whether it shall creep unnoticed and unhonored, buffeted and bruised, along the level of baser elements, or rear a stately trunk, whose wide ascending branches shall adorn with rich verdure the Spring-time of life—bless its Summer with abundant fruit—surround its fading strength with rich perfume, and leave behind lasting evidences of departed glory! If you would attain the power of intellectual manhood, reach the heights of moral sublimity, leave to posterity a legacy in a name—remember, that to the "Know Thyself" of a Pagan Philosophy, a sound, rational, Christian Philosophy adds imperatively, "Govern Thyself." Try every thought, word and action by the standard of God's Word; decide by the light of His counsel; establish early in correct habits head and heart; call to your aid true courage, the noblest characteristic of manhood; moral courage, which will lead and sustain you, often counter to the voice of multitude—often, it may be, bring scoffs and jeers where should be praise; but which will make

> "Your lives sublime,
> And departing leave behind you
> Foot-prints in the sands of Time."

